# A review on the progress of sex-separation techniques for sterile insect technique applications against *Anopheles arabiensis*

**DOI:** 10.1186/s13071-018-3219-4

**Published:** 2018-12-24

**Authors:** Thabo Mashatola, Cyrille Ndo, Lizette L. Koekemoer, Leonard C. Dandalo, Oliver R. Wood, Lerato Malakoane, Yacouba Poumachu, Leanne N. Lobb, Maria Kaiser, Kostas Bourtzis, Givemore Munhenga

**Affiliations:** 1 Centre for Emerging Zoonotic and Parasitic Diseases, National Institute for Communicable Diseases of the National Health Laboratory Service, Johannesburg, South Africa; 2 Wits Research Institute for Malaria, MRC Collaborating Centre for Multi-Disciplinary Research on Malaria, School of Pathology, Faculty of Health Sciences, University of the Witwatersrand, Johannesburg, South Africa; 3 Insect Pest Control Laboratory, Joint FAO/IAEA Division of Nuclear Techniques in Food and Agriculture, Vienna, Austria; 4 Organisation de Coordination pour la lutte Contre les Endémies en Afrique Centrale (OCEAC), Institut de recherche de Yaoundé (IRY), Yaoundé, Cameroon; 5 Department of Biological Sciences, Faculty of Medicine and Pharmaceutical Sciences, University of Douala, Douala, Cameroon; 6 Centre for Research in Infectious Disease (CRI), Yaoundé, Cameroon; 7 Vector Borne Disease Laboratory of the Applied Biology and Ecology Research Unit (VBDL-URBEA) Department of Animal Biology, Faculty of Sciences of the University of Dschang, Dschang, Cameroon

**Keywords:** *Anopheles arabiensis*, sterile insect technique, sex-separation, genetic sexing strain

## Abstract

The feasibility of the sterile insect technique (SIT) as a malaria vector control strategy against *Anopheles arabiensis* has been under investigation over the past decade. One of the critical steps required for the application of this technique to mosquito control is the availability of an efficient and effective sex-separation system. Sex-separation systems eliminate female mosquitoes from the production line prior to irradiation and field release of sterile males. This is necessary because female mosquitoes can transmit pathogens such as malaria and, therefore, their release must be prevented. Sex separation also increases the efficiency of an SIT programme. Various sex-separation strategies have been explored including the exploitation of developmental and behavioural differences between male and female mosquitoes, and genetic approaches. Most of these are however species-specific and are not indicated for the major African malaria vectors such as *An. arabiensis*. As there is currently no reliable sex-separation method for *An. arabiensis*, various strategies were explored in an attempt to develop a robust system that can be applied on a mass-rearing scale. The progress and challenges faced during the development of a sexing system for future pilot and/or large-scale SIT release programmes against *An. arabiensis* are reviewed here. Three methods of sex separation were examined. The first is the use of pupal size for gender prediction. The second is the elimination of blood-feeding adult females through the addition of an endectocide to a blood meal source. The third is the establishment of a genetic sexing strain (GSS) carrying an insecticide resistance selectable marker (dieldrin-resistance *rdl* gene and/or other GABA receptor antagonists that can be used as alternative insecticides to dieldrin) or a temperature-sensitive lethal marker.

## Background

Malaria is a major global health problem responsible for approximately 216 million cases and 445,000 deaths worldwide in 2016 alone [1]. An estimated 80% of malaria-related deaths occur in sub-Saharan Africa, particularly in pregnant women and children below the age of five years. By affecting health at the personal and community levels, malaria also has a direct impact on national economies, education and social development [1, 2].

Various vector control interventions such as larval source management, distribution of insecticide-treated bednets (ITNs) and indoor residual spraying (IRS) of formulated insecticides have contributed significantly towards suppression of the global malaria burden [3–5]. However, the burgeoning incidence of insecticide resistance in target vector populations is threatening control efforts [6–8], as is the diversity of malaria vector species assemblages which often include outdoor-resting and feeding components that are less susceptible to control by indoor applications of insecticide [9, 10]. To address these challenges, supplementary vector control interventions that are effective, environmentally friendly and economical are required. Numerous technologies with potential such as transgenics including gene drive, *Wolbachia*-based methods and the sterile insect technique (SIT) are currently under development or are undergoing open field-testing and validation [11–20].

The SIT has been successfully applied as an area-wide integrated vector management (AW-IVM) method against several insect pests [21, 22]. This technique involves the serial release of laboratory mass-produced sterile insects, usually males, at a ratio that effectively inundates a target wild population. This forces the majority of females to mate with sterile males, substantially reducing their fecundity, and resulting in population suppression [21].

Attempts to use the SIT against mosquitoes dates back six decades [23]. Since then substantial research, answering key questions on mosquitoes and the SIT, has been conducted [11]. For the African malaria vector *An. arabiensis* several research activities are underway in Reunion Island, Sudan and South Africa. Data on the following aspects has been generated: (i) colonization and rearing conditions [24, 25], and (ii) male sterility induction through irradiation and related longevity, mating competitiveness, mating compatibility [24, 26–30], and means of transportation to release localities [24, 26]. In addition to these, field study site selection, and assessments of the monthly species and genetic abundances, and variability of wild populations have been undertaken [31–33]. A study on the technical and social perspectives of using the SIT to control malaria has been conducted [34], including the recent knowledge, attitude and practices (KAP) survey performed in South Africa in an area selected for pilot SIT releases [35]. Before the SIT can be applied at an operational level, there is one critical aspect remaining. This is the development of a robust sex separation system to exclusively obtain males prior to releases [36, 37].

An efficient sex separation system is essential for any mosquito SIT-based control programme for several reasons. The most important reason is that released females have the potential to transmit malaria or other disease pathogens [38], thus putting the human population at risk. Also, the co-release of laboratory females can interfere with the frequency at which sterile males mate with wild females, thus decreasing the effectiveness of the SIT programme. This has been demonstrated in the Mediterranean fruit fly (medfly) where, by releasing males only, the effectiveness of the SIT programme was increased [39, 40].

Several sex separation methods have been developed and implemented in SIT programmes, including mosquito programmes [41, 42]. However, most of these methods are species-specific and are not indicated for *An. arabiensis* or, if applicable, have never been tested at an operational level [42]. Investigations and the development of various robust sexing strategies that can be applied on a mass scale are therefore being conducted in an attempt to address this shortfall. The progress, challenges and lessons learned from these investigations, and future prospects, are reported here.

### Use of pupal size for gender prediction

In many insect species, female pupae are usually larger than their male counterparts [43]. This phenomenon, known as sexual size dimorphism, is a trait that has been exploited for mechanical separation means, with numerous mechanical devices developed for mosquitoes. Examples include standard sieves, the Fay-Morlan glass plate and the McCray adjustable opening separation system that have been used to separate male and female pupae in *Aedes aegypti*, *Ae. albopictus* and *Culex quinquefasciatus* [44–47]*.* Attempts to use sexual size dimorphism in *An. albimanus* were without success during the 1972 sterile male release study in El Salvador [48]. It was observed that *An. albimanus* male and female pupal sizes overlap substantially, thus making efficient sexing by size difficult. To our knowledge, this assumption has never been tested in other anophelines.

Recently, pupal size dimorphism was investigated in a South African *An. arabiensis* strain. Pupal cephalothorax sizes of larvae reared on a standard larval diet were measured and recorded by gender. Cephalothorax size in males averaged 3.64 mm while those of females averaged 3.66 mm. These are not significantly different, as has also been noted in *An. albimanus*. These data are consistent with other reports that pupal size sexual dimorphism is not apparent in anophelines [49]. Compounding this, the results also illustrated a greater variation in pupal size in males compared to females, making the use of sieve and Fay-Morlan glass plate sex-separation technically challenging and even less reliable. It was consequently concluded that this method of sex-separation is not viable for *An. arabiensis*.

### Addition of toxicants to blood meal sources to eliminate blood-feeding females

As only female mosquitoes take blood meals, this auto-segregation is a highly alluring feature that might be exploited by adding a toxicant to a blood meal (blood-spiking) so as to eliminate blood-feeding females. This approach was used in an SIT programme in El Salvador against *An. albimanus* by adding malathion to a citrated bovine blood meal. The result was a 95% elimination of females and 25% male mortality [50]. During this study, adults had to be kept in cages for several days to allow females to mate prior to blood-feeding. It is important to note that although females can take blood meals prior to mating [51], feeding rates, and therefore the efficacy of this selection method, would more likely improve should the blood meal be presented post mating. This extended storing of mosquitoes in cages is problematic because it creates a bottleneck during mass rearing and male sperm are potentially depleted during holding, negatively impacting their mating potential post release [52]. Another concern raised during the El Salvador trial was a high level of male mortality. The reason for high male mortality was assumed to be tarsal contact of males with malathion from blood excreted by females during feeding [53]. This led to investigations of other toxicants such as boric acid, household detergent and dieldrin [54]. Unfortunately, experiments with these also resulted in high male mortality [54]. Subsequently, spinosad and ivermectin, which do not rely on tarsal contact for their mode of toxicity, were tested [54]. Both spinosad and ivermectin eliminated all females from a mixed sex population of *An. arabiensis* within 4 days without causing significant male mortality. At higher concentrations, both toxins eliminated all females within twelve hours but at a significantly high male mortality cost.

Recently, attempts were made to optimize and adapt this strategy as a sex separation method in a South African *An. arabiensis* strain using ivermectin only. During these experiments, difficulties arose relating to blood-feeding success. The South African *An. arabiensis* strain is routinely maintained on blood from anaesthetized guinea pigs and thus did not readily feed on blood spiked with ivermectin using artificial membrane feeders. An investigation of artificial membrane feeding systems and the adaptability of this strain to feed on this system was therefore required. Direct feeding from live guinea pig skin was compared to Hemotek membrane blood-feeding using three different membranes (collagen, pig intestine casing and Parafilm-M). The following parameters were measured to aid in selection of the optimal membrane: feeding success, fecundity and fertility (egg hatch rates). Feeding success averaged 79% in both collagen and pig intestine casing and only 15% in Parafilm-M (unpublished data). Fecundity of females ranged between 23-46 eggs laid per female and there was no difference in fecundity between the three membranes tested (unpublished data). Eggs oviposited by these females hatched at rates ranging from 76% to 92%. This difference was however not statistically significant (unpublished data). Based on these results and considering other factors such as membrane price, thickness and local availability/production level, pig intestine casing was chosen as the optimal membrane and henceforth used to acclimatize the colony to membrane feeding. This paved the way to do baseline studies to explore the use of ivermectin as a blood toxicant to eliminate *An. arabiensis* females for male-only releases.

Subsequent experiments on the use of blood spiked with ivermectin to eliminate females in the South African strain showed that this approach could eliminate females, albeit at a low female elimination rate compared to that reported in Yamada et al. [54]. About 90% of females were eliminated on a single meal after five consecutive days post feeding, while the remainder were eliminated within 10 days. The difference in female elimination rates observed between this study and that of Yamada et al. [54] could be due to several reasons, one of them being the difference in ivermectin formulation. The South African study used ivermectin MK-933 (Sigma, CAS No. 70288-86-7) while Yamada et al. [54] used 1% ivermectin (Virbamec, Virbac Oesterreich GmbH, Vienna, Austria), a formulation that is similar to the one used in veterinary medicine or in mass drug administration studies in vertebrates to kill mosquitoes [55–57]. Another possibility to consider is that the South African *An. arabiensis* strain has an intrinsic metabolic insecticide resistance mechanism in place [58]. If placed under insecticide selective pressure, the strain has potential to trigger this resistant genotype. It is therefore conceivable that the metabolic detoxification system in this strain, although it is phenotypically insecticide susceptible, could inadvertently have reduced its susceptibility to ivermectin, making female elimination by the spiked blood less effective. Further investigations would be required to test these hypotheses.

Another concern observed during the South African ivermectin optimization trial was a high level of male mortality (21%) when spiked blood meals were presented serially to enhance female elimination. This was however not surprising as similar results have been reported [54] whereby increased tarsal contact with insecticide from blood excreted by females is the likely cause of male mortality. A high rate of male loss prior to releases would adversely affect production efficiency during large-scale SIT operations.

An additional disadvantage of using blood-spiking to eliminate females is the effect on the reproductive capacity of males. The potential loss of male mating capacity as a result of potential exposure to ivermectin was investigated. This was achieved by mating the males that survived post 100% female elimination with untreated virgin females and then dissecting their spermathecae to determine insemination rates. High insemination rates (> 95%) were recorded, with no statistically significant difference found in comparison to virgin males and females that had not been previously exposed to ivermectin, suggesting that ivermectin treatment did not negatively impact male fitness (unpublished data).

Although these results show that ivermectin can be used as a blood toxicant for sex separation, factors such as the inability to reliably guarantee complete elimination of females within a short period have to be addressed before this method can be applied on a mass-rearing scale. Possible improvements could involve improving blood-feeding success, particularly of females that opt not to feed or ingest sufficient quantities of spiked blood. This can be achieved by the addition of phago-stimulants (e.g. adenosine triphosphate (ATP) [59], L-lactic acid [60], or natural odor ligands from human skin [61]) and other attractants into or near the blood. Another possible approach, given that this method is based on auto-segregation of sexes based solely on blood-feeding behaviour, may be to use any nonblood-based stimulus to attract females away from males followed by electrification to kill the females. Additionally, the use of artificial diets [62, 63] can be investigated as they would allow manipulation of ingredients and possible improvements in the efficacy of toxicants. Investigation into artificial diets would contribute positively to both routine colony maintenance and blood-spiking studies.

### Development of a genetic sexing strain (GSS)

Genetic sexing strains (GSSs) are based on the linkage of a selectable marker to a sex-determining chromosome [64]. GSSs have been developed in many insect species, particularly in major agricultural pests such as the medfly *Ceratitis capitata*, the Mexican fruit fly *Anastrepha ludens*, the Oriental fruit fly *Bactrocera dorsalis* and the melon fruit fly *Zeugodacus* (*Bactrocera*) *cucurbitae* [65–69]. In all of these insects, the construction of a GSS involved a mutation that can be used as a selectable marker for sex separation and a Y-autosome translocation linking the inheritance of the wild type allele to the Y chromosome. This results in females carrying the mutant allele and males hemizygous for the wild type. Currently, only the *C. capitata* GSSs are being used at an operational level for mass-rearing [70–72].

The usefulness of Y-autosome translocation based GSSs may be hindered by genetic instability resulting from pre-meiotic recombination in the parental male [73] and/or the survival of genetically unbalanced individuals resulting from simultaneous segregation of non-homologous centromeres (adjacent-1 segregation) during meiosis in the parental males [71]. Recombination events can occur between the translocation break point and the selectable marker, which may result in the accumulation of recombinants and the collapse of the genetic sexing character of the strain [72]. Reduction and/or elimination of recombination can be achieved by the selection of a Y-autosome translocation where the autosomal breakpoint is close to the selectable marker or by the induction of a chromosomal inversion that includes the region of the translocation breakpoint and the selectable markers [71]. In addition, a Filter Rearing System (FRS) can be adopted to remove recombinants that might possibly accumulate during mass-rearing [71, 74, 75]. This would guarantee that the genetic purity of the mass-reared GSS is kept intact. However, a FRS can better work in conjunction with a visible mutation.

Several genetic/selectable markers exist in anophelines [76–84]. However, most of them have not been assessed in respect to their potential use in the construction of a GSS. The most promising or stable selectable markers successfully used to date for the development of GSSs in insects include insecticide resistance [85] as well as color- and temperature-sensitive lethal mutations (*tsl)* [71]. GSS based on insecticide resistance markers have already been developed in *An. arabiensis* while a *tsl*-based GSS is currently under development [36, 83, 85].

### Insecticide resistance based GSS

Several GSSs based on insecticide resistance markers have been developed in a number of anophelines, including *An. gambiae* (s.s.) [64], *An. albimanus* [86], *An. stephensi* [87], *An. quadrimaculatus* [88] and *An. arabiensis* [89, 90]. Of these, only *An. albimanus* has been used on a mass-rearing scale [91]. The drawback of using insecticides as selectable markers for sex separation during mass-rearing for SIT applications is the negative impact to the environment caused by accidental release of insecticides during treatment to eliminate females, risk of insecticide contaminating a mass-rearing colony, genetic instability of the marker and maintenance difficulties due to inherent sterility caused by chromosomal translocations [92].

Owing to a scarcity of other alternative selectable markers, the organochloride insecticide, dieldrin, was recently used to develop an *An. arabiensis* GSS ANO IPCL1 strain that allowed separation of males from females [85]. Life history characteristics such as egg hatch rates, development, longevity, female elimination reliability, radiation sensitivity and mating competitiveness were evaluated as part of efforts to establish a strain that can be useful in mass rearing [93–95]. Apart from high semi-sterility (73%) in males from the ANO IPCL1 strain, no differences in life history traits were found when compared to a dieldrin susceptible strain [94]. Tests on the genetic stability of the strain showed recombination rates to be as low as ∼0.4%, lower than previously reported [96], making it favorable for mass-rearing. An added advantage that was observed when using dieldrin to separate males from females was the possible synergistic effect of the dieldrin whereby irradiated male pupae from dieldrin treated eggs continued to produce sperm in the first week of adult life, while adult males that had only been irradiated as pupae without the dieldrin treatment ceased to produce sperm. The advantage of this is that, from a sterile male release perspective, these males are expected to maintain their mating vigour post-release [51]. This led to the hypothesis that dieldrin treatment might have a protective effect on the germinal cells of *An. arabiensis* against radiation [96]. Despite all these advantages, the use of the strain is threatened by environmental concerns. Dieldrin use for field research and applications has been prohibited since the 1970’s. In addition, recent studies showed that dieldrin adhered to treatment containers and treated eggs retained residual dieldrin until adulthood following absorption through the chorion [97], and the overuse of dieldrin could aggravate the ever-present risk of potential contamination of other non-targeted colonies in the laboratory [95]. Lastly, the stability of this strain requires further verification as the low recombination rates observed in Yamada et al. [96] were based on phenotypic expression of the insecticide selectable marker rather than genetic monitoring.

Considerations need to be taken relating to the genetic background of a GSS and its mating compatibility with mosquitoes of a different geographic location for SIT purposes. Until recently, the only available *An. arabiensis* GSS was the ANO IPCL1 that comprised a Sudanese genetic background and as such may not be directly used for SIT releases in other countries. The introduction of an exogenous mosquito strain may face difficulties, such as possible mating incompatibility, that will dramatically affect the efficiency of SIT applications, regulatory approval, ethical concerns of releasing mosquitoes from a different geographical region and public acceptance. The South African SIT programme addressed these concerns by introgressing the dieldrin resistant gene from GSS ANO IPCL1 males into a locally colonized *An. arabiensis* wild-type strain (acronym KWAG), therefore maintaining a locally representative genetic background in the resultant new GSS strain (acronym GMK) [36].

The strain initially showed a reduction in egg hatch rates following repeated treatment with dieldrin at each generation (about 19.2%), which then improved to 30% with successive backcrossing [37]. The dieldrin resistance marker of GMK has been stable over 10 successive generations [37]. Tests for the presence or absence of the resistance to dieldrin (*Rdl*) mutation showed that 100% of the males were hemizygous for the resistant allele and 100% of the females were homozygous for the susceptible allele [37]. A high mating competitiveness against wild-type males when in competition for wild females was also observed in GMK males [37]. Additionally, the effect of irradiation on GMK females was investigated and compared to unirradiated females showing a negative effect of irradiation on female adult emergence [98]. However, GMK females were still capable of blood-feeding and demonstrated no difference in longevity post-irradiation. This result illustrates the importance for sex separation in mosquito SIT programmes because these females could potentially transmit pathogens during blood-feeding. Additionally, GMK is showing similar problems as observed in GSS ANO IPCL1, such as low productivity, dieldrin adherence to containers and absorption through the chorion. Due to these problems investigations of alternative, more environmentally acceptable, insecticides are taking place.

Theoretically, insecticides with a similar mode of action to dieldrin, i.e. targeting the γ-aminobutyric acid (GABA) receptor, should be able to be used as substitutes to dieldrin [99]. Several insecticides (lindane, picrotoxin, isoxoxale) which target the GABA receptor were tested. Treating third and fourth instar GMK larvae with lindane and picrotoxin eliminated 90% of the females showing that any insecticide that solely targets the GABA site can act as an alternative to dieldrin. Further investigations are currently ongoing to exploit plant based/organic alternatives (unpublished data).

### Temperature-sensitive lethal based GSS

Temperature sensitive lethal mutations have been used successfully as sexing system selectable markers for *C. capitata* where females that are homozygous for the recessive mutation die when exposed to high temperatures, while hemizygous males survive under the same conditions [71, 100]. This is probably because the *tsl* mutation alters the function of an essential protein at different temperatures, the practical upshot of which is that at regular rearing temperatures protein function is maintained, but at higher temperature restrictive protein function is lost [100]. Other than the financial savings in not using insecticides and avoiding environmental or equipment contamination, *tsl-*based GSS also has the added advantage of the removal of females at a very early developmental stage (during embryonic stage). This translates to rearing cost reduction [71].

Initiatives are currently underway to develop a *tsl*-based GSS in *An. arabiensis* [42, 83]. Progress made thus far includes the successful isolation and characterization of an *An. arabiensis tsl* [83] following similar methods as [101]. During these efforts, wild-type male mosquitoes originating from North Cameroon were provided with a sugar solution spiked with 0.05M of ethyl methanesulfonate (EMS), for 24 hours [83]. Mutant male mosquitoes were crossed with virgin wild-type females and third generation (F3) progeny were heat-shocked at 41°C for 3 hours to screen for *tsl*. The established *tsl* strain showed similar life history traits (fertility, larval development time and adults’ emergence) compared to the wild-type strain, and can be maintained at the same rearing temperature, i.e. 26 ± 1°C, as the wild-type strain. Preliminary genetic analysis suggests that the *tsl* phenotype is due to a recessive allele located on an autosome [83].

The successful establishment of the *An. arabiensis tsl* strain is a valuable tool towards the development of a GSS for SIT applications against this species. Future research will focus on the characterization of the temperature-sensitivity range, the induction of a Y-autosome translocation to link the wild-type allele to the Y chromosome as well as the identification and characterization of the *tsl* gene and its potential use for novel approaches to develop a GSS for this species.

The characterization of the temperature-sensitive period is important as the temperature sensitivity status of strains differs depending on the developmental stage, duration of exposure and their insecticide resistance status [102–110]. Tests on the temperature sensitivity range (permissive and restrictive) should be performed to aid in establishing optimal rearing conditions, particularly under mass rearing settings. This can be achieved by exposing various developmental stages (from embryos to adults) to different temperatures and time ranges with an emphasis on the embryonic stage as this is the most practical stage [71].

The induction of a Y-autosome translocation will enable establishment of families where males are wild-type (temperature resistant) and females are mutant (temperature sensitive). Irradiation can be used to translocate alleles to the male determining Y chromosome [111, 112]. This can be followed by determination of the inheritance pattern to confirm that *tsl* is indeed inherited in a sex-specific manner.

Isolation of a traceable selectable marker linked to the *tsl* will allow for tracking of recombinants in mass-rearing systems [71]. In a *tsl*-based GSS, it would almost be impossible to detect and remove the recombinants without a traceable selectable marker [71]. A selectable marker that is close to the *tsl* locus would most likely be a good candidate, especially in the case of a visible selectable marker, as has been the case with the medfly VIENNA 8 GSS which carries a visible *white pupae* (*wp*) marker closely linked to the *tsl* on chromosome 5 [72].

**Fig. 1:**
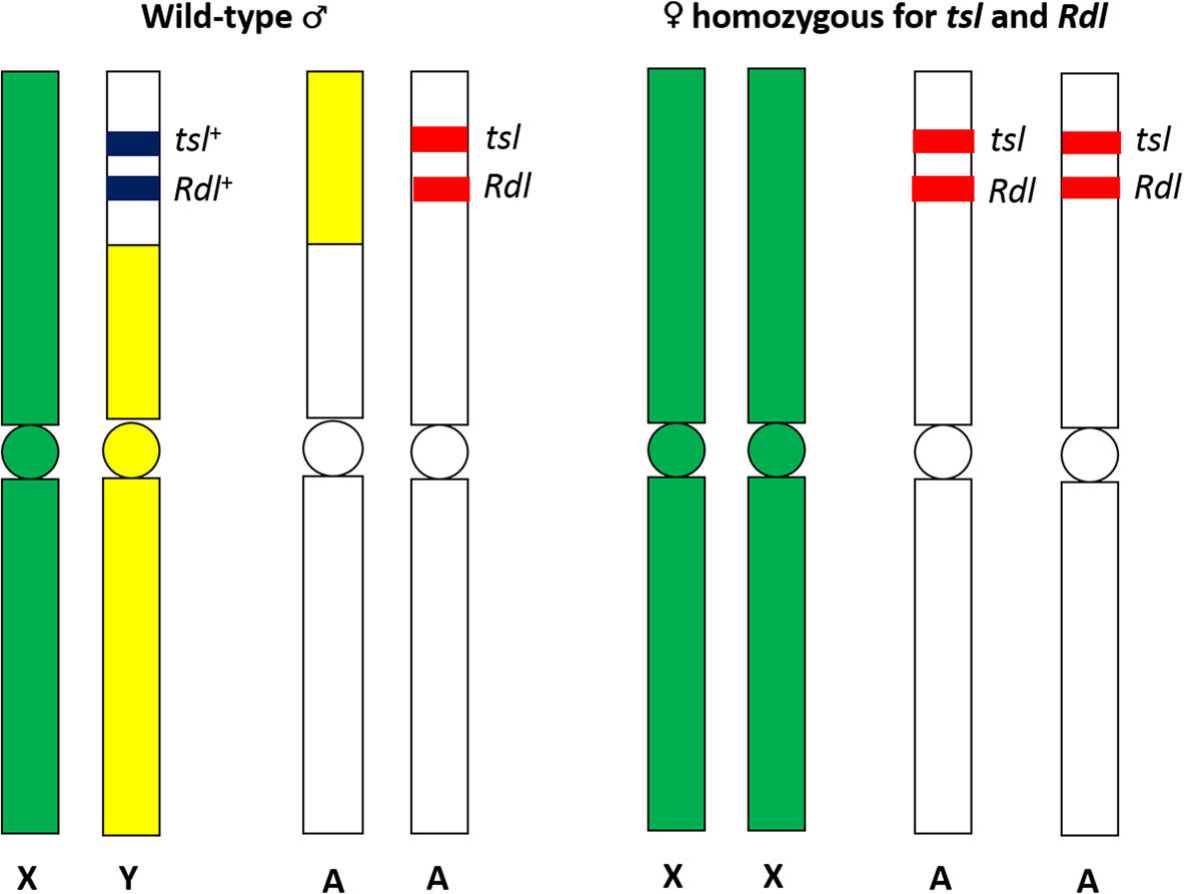
A schematic diagram showing how traceable phenotypic selectable markers such as *Rdl* could be used in conjunction with *tsl*, especially if they are closely linked on the same chromosome. In a GSS chromosomal translocation involving *tsl* and *Rdl*, males are hemizygous for the wild-type alleles of both loci (marked as blue) which are translocated to the Y chromosome (shown predominantly in yellow), with a reciprocal translocation to the autosome. The remaining, intact autosome carries the mutant alleles of both *tsl* and *Rdl* loci (marked as red). Females, which lack the Y chromosome, are homozygous for the mutant alleles of both *tsl* and *Rdl*, conferring the mutant phenotypes that allow for male selection, i.e. treatment with either temperature or dieldrin kills females (♀ = female, ♂ = male).

There are several *tsl* loci in a genome; however, traceable phenotypic selectable markers such as *Rdl* may possibly be used in conjunction with *tsl* in a *tsl*-based GSS (Fig. 1). This hypothesis was drawn against reported findings in *Drosophila melanogaster* which showed *Rdl* and *tsl* to occur at the same locus [113, 114], leading to the hypothesis that this might also be true in other insects, including mosquitoes. Exploiting the *Rdl* locus in *An. arabiensis* may be promising because it has already been proven to induce conditional lethality [36, 85], both dominant and semi-dominant *Rdl* alleles are known [115], which allows for easy discrimination of homozygous susceptible and heterozygous resistant larvae and/or adults using a diagnostic dose of dieldrin. The mechanism of resistance is also known to be due to a single amino acid substitution in the target site [116], and the loci can easily be detected using polymerase chain reaction (PCR) [117].

Since multiple insecticide resistance and cross-resistance [118–123] together with trade-offs between temperature and insecticide resistance have been reported in various studies [102, 105, 106, 108, 110], it can be hypothesized that these markers possibly behave in a similar fashion as *Rdl* and also could occur at the same loci as *tsl*. Therefore, investigations into insecticide resistance markers should be extended to include other target sites, including the knockdown resistance gene (*kdr*) [124–126], the acetylcholinesterase gene (*ace*-*1*^*R*^) mutation [127–130] and molecular gene markers [131–133]. Alternatively, EMS can be used to induce other mutations as has been previously reported for three new *An. quadrimaculatus* mutants: (1) *rose eye* (*ro*), (2) *short antenna* (*Sa*) and (3) *melanotic* (*Mel*) [134–136]. The advantage of this approach is that a new GSS based on color can also be established.

Additionally, as the efficiency of a *tsl*-based GSS relies on the genetic composition of the sex chromosomes, knowledge on the cytogenetics of the target species will also be necessary. Fortunately, detailed reports exist on the *Anopheles* Y chromosome, which constitute essential components necessary for sex separation [137–139]. Cytogenetics can aid in the determination of the origin and size of the translocated segment, and localization of the translocation break-points [140–142], or map the extent of inversions introduced to reduce recombination [143, 144]. To maintain stability and avoid high recombination rates, a chromosomal inversion, ideally covering the region of translocation break-point, the *tsl,* and any other selectable markers, could also be integrated in the GSS. Inversions are known suppressors of genetic recombination through a positive heterotic system. In fact, maintenance and stability of dieldrin resistance in *An. gambiae* is associated with a paracentric inversion, 2La [145] and this could greatly improve the genetic stability of the GSS [71, 72, 143]. This has been shown in the medfly VIENNA 8 GSS [71]. Cytogenetic analyses such as karyotype examination of mitotic and meiotic chromosomes [146] and salivary gland polytene chromosomes [147] can be performed to map mutations and identify Y-autosome breakpoints in the resultant Y-autosome translocations/GSS. Subsequent investigations into life history traits together with genetic characterization needed prior to the strain’s use in large-scale operational SIT programmes [71].

GSSs can also be developed using novel molecular-based approaches including, among others, conditional lethal systems, RNAi approaches or by using transgenic strains with integrated sex-specific fluorescent markers [41, 148, 149].

## Conclusions

The development of a sex separation strategy using pupal sexual dimorphism is not applicable to *An. arabiensis*. The use of ivermectin to eliminate females shows great potential, but currently cannot be relied upon as a sole sex-separation strategy and would require further investigation on formulations and effectiveness if added to an artificial blood-meal. The practicality of using ivermectin in mass-rearing will also need to be tested. Alternatively, research can be focused on exploiting female blood-seeking behaviour without using blood at all but simply as a segregation tool. Furthermore, male behaviour though not fully explored in this review has potential. It has been suggested that sex-specific male swarming behaviour in anophelines could be exploited to develop efficient sex separation strategy. The success achieved in developing a GSS containing a South African genetic background and positive attributes such as accelerated development of aquatic stages and high survival rates at all life stages has provided encouragement for the application of this GSS in the local SIT pilot studies, unless a suitable alternative to dieldrin-based sex-separation can be found. Further investigations and optimization of treatment procedures and monitoring of the genetic stability of the strain are still required. The promising results shown by some alternate insecticides may provide additional options and solutions to the difficulties faced by the current dieldrin-based GSS. The successful establishment and characterization of an *An. arabiensis tsl* strain offers possibilities into development of a new GSS. In its current form, the *tsl* strain cannot be directly applied as a GSS and would require the characterization of the temperature-sensitivity range, inheritance linkage of the wild-type allele to the sex determining Y chromosome and isolation of a visible selectable marker closely linked to *tsl*. If the linkage of *tsl* and *Rdl* in Drosophila also exists in *An. arabiensis*, this would allow the easier monitoring of the *tsl* marker until a better, ideally visible, marker linked to *tsl* is isolated. This can be achieved through the screening of laboratory and natural populations for spontaneous mutations or EMS and/or irradiation-induced mutagenesis. Finally, knowledge on the cytogenetics of the target species will be necessary and subsequent investigations into life history traits together with genetic characterization are warranted prior to the strain being used in large-scale operational SIT programmes.

## Abbreviations

ACT: Artemisinin-based combination therapy
ATP: Adenosine triphosphate
EMS: Ethyl methanesulfonate
GABA: γ-aminobutyric acid
GSS: Genetic sexing strain
IRS: Indoor residual spraying
ITNs: Insecticide-treated bednets
*Rdl*: Resistance to dieldrin
SIT: Sterile insect technique
*tsl*: Temperature-sensitive lethal
